# Cardiac troponins predict mortality and cardiovascular outcomes in patients with peripheral artery disease: A systematic review and meta‐analysis of adjusted observational studies

**DOI:** 10.1002/clc.23776

**Published:** 2022-02-07

**Authors:** Mislav Vrsalovic, Ana Vrsalovic Presecki, Victor Aboyans

**Affiliations:** ^1^ Department of Internal Medicine University of Zagreb School of Medicine Zagreb Croatia; ^2^ Department of Cardiology Sestre Milosrdnice University Hospital Center Zagreb Croatia; ^3^ Department of Reaction Engineering and Catalysis, Faculty of Chemical Engineering and Technology University of Zagreb Zagreb Croatia; ^4^ Department of Cardiology Dupuytren University Hospital Limoges France; ^5^ INSERM 1094 Limoges University Limoges France

**Keywords:** critical limb ischemia, major cardiovascular events, meta‐analysis, mortality, peripheral artery disease, troponin

## Abstract

**Background:**

A significant proportion of patients (pts) with peripheral artery disease (PAD) have concomitant coronary artery disease and polyvascular involvement contributes to increased risk of death and unfavorable cardiovascular events.

**Hypothesis:**

Cardiac troponins are associated with adverse cardiovascular outcomes in PAD pts.

**Methods:**

We systematically searched Medline and Scopus to identify all observational cohort studies published before June 2021 (combining terms “troponin,” “peripheral artery disease,” “peripheral arterial disease,” “intermittent claudication,” and “critical limb ischemia”) that evaluated the prognostic impact of troponin rise on admission on all‐cause mortality and/or major cardiovascular events (MACEs; composite of myocardial infarction, stroke, and cardiovascular death) in PAD pts followed up at least 6 months. A meta‐analysis was conducted using the generic inverse variance method. Heterogeneity between studies was investigated using Cochrane's Q test and *I* ^2^ statistic.

**Results:**

Eight studies were included in the final analysis (5313 pts) with a median follow‐up of 27 months (interquartile range: 12–59 months). The prevalence of troponin positivity was 5.3% (range: 4.4%–8.7%) in pts with intermittent claudication, and 62.6% (range: 33.6%–85%) in critical limb ischemia. Elevated troponins were significantly associated with an increased risk of all‐cause mortality (hazard ratio [HR]: 2.85, 95% confidence interval [CI]: 2.28–3.57; *I* ^2^ = 50.97%), and MACE (HR: 2.58, 95% CI: 2.04–3.26; *I* ^2^ = 4.00%) without publication bias (*p* = .24 and *p* = .10, respectively).

**Conclusion:**

Troponin rise on admission is associated with adverse long‐term cardiovascular outcomes in symptomatic PAD.

## 1 INTRODUCTION

Peripheral artery disease (PAD) is one of the most severe manifestations of atherosclerosis, and due to aging of the population and increase in traditional cardiovascular risk factors PAD will become more common in the future.^1^ Moreover, PAD is a marker of a more advanced disease process, affecting often multiple vascular beds, and up to 70% of patients with PAD may have concomitant coronary artery disease (CAD).^2^, ^3^ This polyvascular involvement contributes to an increased risk of death and adverse cardiovascular events.^3^ Screening for CAD in PAD patients may be useful for risk stratification, as morbidity and mortality are mostly of cardiac origin. However, noninvasive evaluation of CAD (i.e., treadmill stress test, coronary computed tomography angiography) is limited in this patient population due to the inability of exercise testing, and frequent occurrence of diffuse arterial calcifications. In the current guidelines, screening for the other sites of atherosclerosis, including routine coronary angiography, to improve outcomes in patients with lower extremity PAD was considered as one of the main gaps in evidence in the management of patients with PAD that needs further investigation.^2^

Circulating biomarkers are attractive tools in diagnostic decision making and risk stratification in various cardiovascular conditions.^4^ Although, cardiac troponins are widely used in routine cardiology clinical practice its independent prognostic interest in PAD has not yet been systematically investigated.^4^, ^5^ Troponin level rise may identify high‐risk PAD patients who could benefit from individualized screening for multisite artery disease and novel treatment strategies (i.e., antithrombotic, lipid‐lowering, and antidiabetic therapies).^6^

Therefore, we performed a systematic review and meta‐analysis to evaluate whether troponin rise at admission was associated with an increased risk of all‐cause mortality and/or major cardiovascular events in patients with symptomatic PAD.

## 2 PATIENTS AND METHODS

### 2.1 Search strategy

This meta‐analysis was performed in accordance with the PRISMA statement.^7^ We performed a systematic literature search of Medline and Scopus for all studies published before June 2021 without language restriction, using the following medical subject headings: “troponin,” “peripheral artery disease,” “peripheral arterial disease,” “intermittent claudication,” and “critical limb ischemia.” Additional studies were identified by manual search of references of original studies or review studies. Ethical approval or patient consent were not required because this study retrieved and synthesized data from already published studies.

### 2.2 Study inclusion and outcomes

We included observational cohort studies that evaluated the prognostic impact of troponin elevation (positivity) on admission on all‐cause mortality and/or major cardiovascular events (MACEs, composite endpoint of myocardial infarction, stroke, and cardiovascular death) in patients with PAD with a follow‐up period of at least 6 months. The studies included symptomatic PAD patients (intermittent claudication and/or critical limb ischemia [CLI]) and the diagnosis was made according to the ankle‐brachial index measurement (<0.9 indicating disease). All patients were categorized based on the presence or absence of troponin elevation at the time of enrollment and clinical outcomes of troponin positive were compared with troponin negative patients. High‐sensitivity cardiac troponins were reported in the included studies and the 99th percentile value of a reference population was chosen to define an elevated troponin level.

### 2.3 Data extraction and quality assessment

Study selection and data extraction were conducted independently by two investigators (M. V. and A. V. P.). Any disagreements or differences in the data extraction between the two authors were harmonized by consensus after rechecking the source data. Study quality was assessed using the validated Newcastle‐Ottawa Scale for assessment of nonrandomized and observational studies, and studies were evaluated based on subject selection, comparability of study groups, and assessment of the outcome.^8^ Risk of bias was evaluated for each study according to the ROBINS‐I tool.^9^ Completed database contained the following data: name of the first author, year of publication, country of origin, the total number of patients in each study, mean age of a population, the percentage of males, study design, troponin cutoff value and diagnostic assay, percentage of patients with elevated troponin levels, the proportion of patients with hypertension, diabetes, CAD, and CLI, the use of antiplatelet drugs and statins, the percentage of patients who died and experienced MACE, the follow‐up period, adjusted hazard ratios (HRs), and confounding factors.

### 2.4 Statistical analysis

A meta‐analysis was conducted using the generic inverse variance method. Multivariable adjusted HRs were used to determine the strength of an association with each endpoint. The estimates of log HRs and standard errors have been obtained from the results of Cox proportional hazards regression models of the included studies. Heterogeneity between studies was investigated using the Cochrane's Q test and *I* ^2^ statistic. Statistically significant heterogeneity was considered present at *p* < .10 and *I* ^2^ > 50%. Sensitivity analyses were performed by excluding studies one at a time to test the contribution of each study to the pooled estimates. Publication bias was assessed graphically using a funnel plot. Analyses were conducted using MedCalc Statistical Software version 20.011.

## 3 RESULTS

### 3.1 Selected studies and baseline characteristics

**Figure 1:**
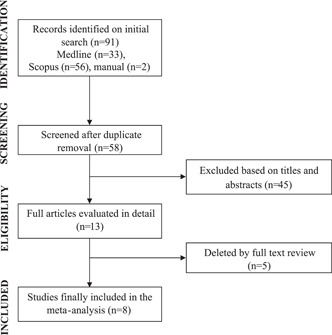
Study flow diagram for meta‐analysis of cardiac troponins and all‐cause mortality and/or cardiovascular outcomes in patients with symptomatic peripheral artery disease

Overall, 91 documents were initially identified from electronic search. After reading titles and abstracts, followed by a review of potentially relevant studies, eight studies were included in the final analysis, including a total of 5313 patients (Figure 1).^10^, ^11^, ^12^, ^13^, ^14^, ^15^, ^16^, ^17^ Seven studies were prospective,^10^, ^11^, ^12^, ^13^, ^15^, ^16^, ^17^ and one was retrospective.^14^ The study characteristics are listed in Table 1. Using the Newcastle–Ottawa scoring system, for included studies median score was 8. Risk of bias was reported for each study according to ROBINS‐I tool (Supporting Information Table). The median age of the population was 71 years (interquartile range [IQR]: 65–73 years), 73% were males, 76% had hypertension, 57% had CAD, 40% had diabetes, and 80% were on statin therapy. The prevalence of patients with CLI was 27%. A median follow‐up period was 27 months (IQR: 12–59 months). Multivariate statistical analysis was performed in all the studies.

**Table 1:** Characteristics of studies included in the meta‐analysis

Author, year, country	Pts (*n*)	Age (years)	Male (%)	Follow‐up (months)	Design	HTN (%)	CAD (%)	DM (%)	CLI (%)	Troponin positive (%)	Assay	Cutoff (ng/ml)	Antiplatelet (%)	Statins (%)	MACE (%)	Mortality (%)	Adjusted HR (95% CI)	Confounders	NOS (0–9)
Spark, 2010, Australia, UK, Canada	149	73	NR	9	P	56	9.4	30	100	33.6	Bayer Diagnostics, Tarrytown, NY	0.1	NR	26	–	43.4	3.1 (1.6–5.6)	Age, smoking, CAD, CVD, renal failure, statins, NLR	8
Linnemann, 2014, Germany	1041	71	63	12	R	85	45	41	40	21.3	Elecsys 6000 system, Roche Diagnostics	0.01	97	72	–	10	4.64 (2.82–7.64)	Age, GFR, smoking, DM, CLI	8
Pohlhammer, 2014, Austria	235	58	100	84	P	86	29	16	0	6.4	5th Generation, Modular, Roche Diagnostics	0.014	NR	NR	14	16	5.06 (2.23–10.85)	Age, GFR, smoking, CRP, CAD	8
																	3.25 (1.30–8.15)^a^		
Otaki, 2015, Japan	208	74	84	23	P	83	31	47	10	NR	Elecsys 2010 System, Roche Diagnostics	0.016	70	50	19	–	2.14 (1.42–3.23)^a^	CLI, CAD, CKD	8
Eisen, 2017, TRA2P ‐ TIMI 50	2728	62	75	30	P	70	74	26	0	4.4	Abbott ARCHITECT	0.026	92	88	19	–	5.43 (2.32–12.72)^a^	Age, sex, DM, HTN, GFR, hyperlipidemia smoking, CAD, heart failure, BNP, CRP,	8
Szczeklik, 2018, Poland	239	71.5	56	12	P	77	48	58	100	62	Elecsys 2010 System, Roche Diagnostics	0.014	98	87	20.5	14.2	2.44 (1.18–5.06)	Sex, CAD, DM, Rutherford grade, GFR, BNP	8
																	2.89 (1.41–5.92)^a^		
Clemens, 2019, Switzerland	95	68	73	114	P	81	23	32	0	NR	COBAS 8000, Roche Diagnostics	0.01	NR	NR	–	46	1.93 (1.33–2.79)	Age, sex, DM, CVD	8
Cimaglia, 2021, Italy	618	73	72	33	P	88	44	100	100	85	Cobas e601 Roche, Diagnostics	0.014	93	79	29	25	2.90 (1.55–5.43) 2.44 (1.71–3.49)^a^	GFR, heart failure. BMI, hemoglobin, CAD, CRP, atrial fibrillation, statin, stroke, COPD	8

Abbreviations: BMI, body mass index; BNP, brain natriuretic peptide; CAD, coronary artery disease; CI, confidence interval; CKD, chronic kidney disease; CLI, critical limb ischemia; COPD, chronic obstructive pulmonary disease; CRP, C‐reactive protein; CVD, cerebrovascular disease; DM, diabetes mellitus; GFR, glomerular filtration rate; HR, hazard ratio; HTN, hypertension; MACE, major adverse cardiovascular event; NOS, Newcastle–Ottawa score; NLR, neutrophil/lymphocyte ratio; NR, not reported; Pts, patients; TRA2P ‐ TIMI 50, trial to assess the effects of vorapaxar in preventing heart attack and stroke in patients with atherosclerosis.

^a^ MACE.

Studies reported variable rates of increased troponin levels, ranging from 4.4% in claudicants up to 85% in patients with CLI. In a subgroup of patients with intermittent claudication the overall prevalence of troponin positivity was 5.3% (range: 4.4%–8.7%), while 62.6% (range: 33.6%–85%) of CLI patients had elevated troponin levels.

### 3.2 Quantitative data synthesis

**Figure 2:**
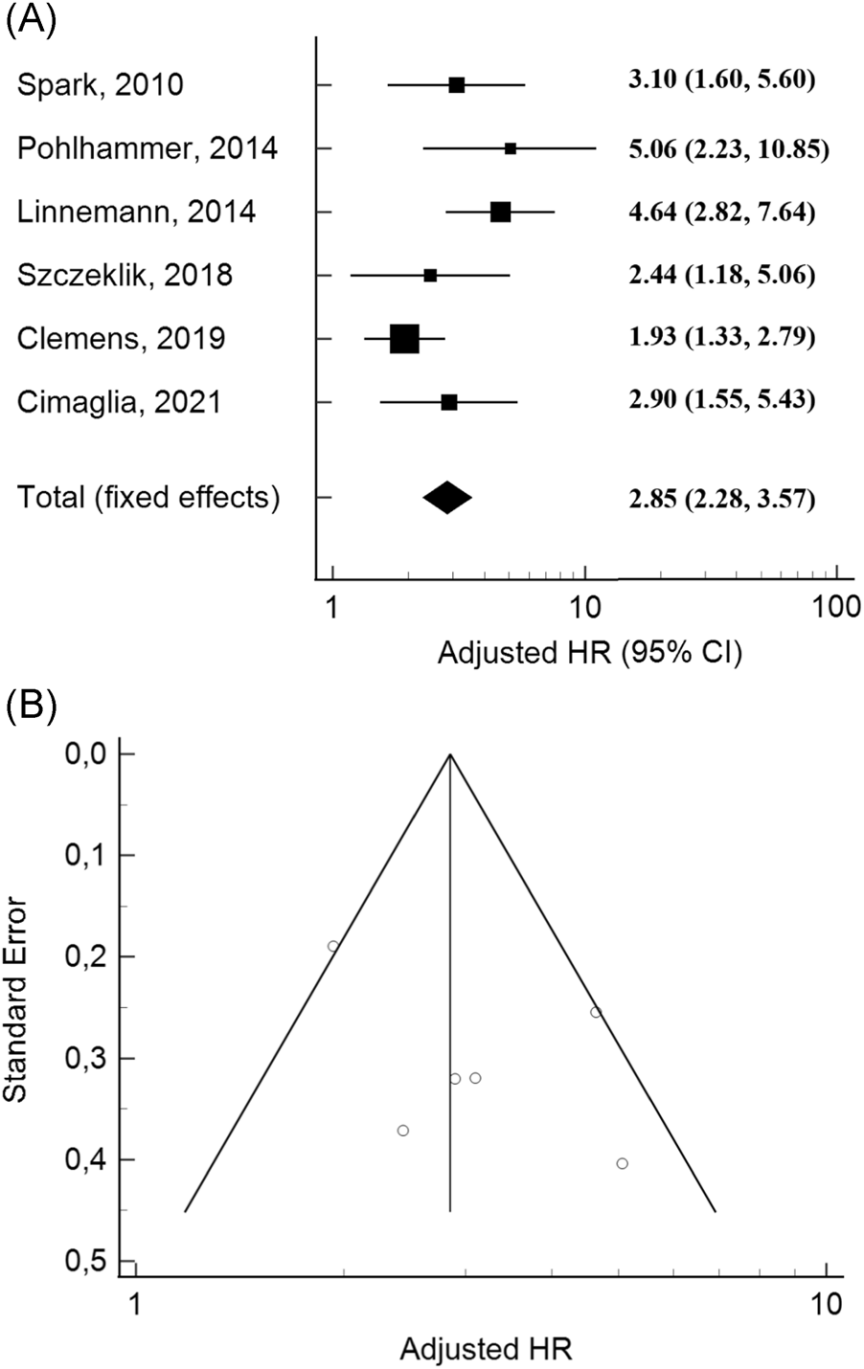
Forest plots of adjusted hazard ratios (HRs) for baseline cardiac troponin to predict long‐term mortality in peripheral artery disease. (A) Funnel plot of adjusted HRs showing no publication bias (*p* = .24). (B) The meta‐analysis was conducted using the generic inverse variance method, and pooled HR was reported with 95% confidence interval (CI)

In pooled analysis, there was a significant association between elevated troponin levels and all‐cause mortality (HR: 2.85, 95% confidence interval [CI]: 2.28–3.57) (Figure 2A). The analysis of pooled studies showed a moderate heterogeneity (*I* ^2^ = 50.97%, Cochran *Q* = 10.20, *p* = .07), without evidence of publication bias (Egger's test: *p* = .24; Figure 2B). Sensitivity analyses revealed that one study^15^ had a significant impact on between‐study heterogeneity (Table 2).

**Table 2:** Sensitivity analyses excluding one study at a time

Author, year	MACE			Mortality		
	HR	95% CI	*I* ^2^ (%)	HR	95% CI	*I* ^2^ (%)
Spark, 2010	–	–	–	2.82	2.21–3.58	60.47
Linnemann, 2014	–	–	–	2.52	1.96–3.24	28.50
Pohlhammer, 2014	2.54	1.99–3.23	23.16	2.71	2.15–3.43	50.00
Otaki, 2015	2.82	2.12–3.75	0.11	–	–	–
Eisen, 2017	2.42	1.90–3.09	0.00	–	–	–
Szczeklik, 2018	2.54	1.98–3.26	26.05	2.90	2.29–3.67	60.02
Clemens, 2019	–	–	–	3.57	2.70–4.73	0.00
Cimaglia, 2021	2.69	1.97–3.66	25.19	2.84	2.24–3.62	60.77

Abbreviations: CI, confidence interval; HR, hazard ratio; MACE, major adverse cardiovascular event.

**Figure 3:**
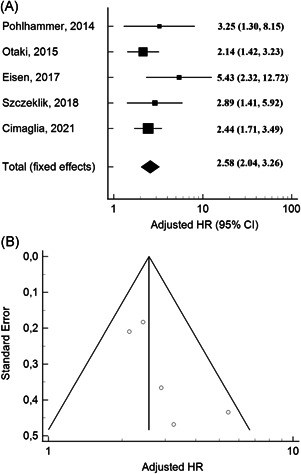
Forest plots of adjusted hazard ratios (HRs) for baseline cardiac troponin to predict long‐term major cardiovascular events (myocardial infarction, stroke, and cardiovascular death) in peripheral artery disease. (A) Funnel plot of adjusted HRs showing no publication bias (*p* = .10). (B) The meta‐analysis was conducted using the generic inverse variance method, and pooled HR was reported with 95% confidence interval (CI)

Meta‐analysis of studies that reported MACEs showed a significant impact of troponin positivity on cardiovascular outcomes (HR: 2.58, 95% CI: 2.04–3.26) (Figure 3A). A low heterogeneity was observed across the studies (*I* ^2^ = 4.00%, Cochran *Q* = 4.17, *p* = .38), without publication bias (Egger's test: *p* = .10; Figure 3B).

Three studies^10^, ^11^, ^17^ investigated outcomes exclusively in patients with CLI including 1006 patients. Meta‐analyses showed a significant association between elevated troponin levels and all‐cause mortality (HR: 2.84, 95% CI: 1.94–4.14; *I* ^2^ = 0.00%) and MACE (HR: 2.52, 95% CI: 1.83–3.48; *I* ^2^ = 0.00%) with low heterogeneity between studies.

Only one study investigated the amputation risk according to troponin increased level.^14^ Peripheral vascular patients with elevated troponins had an increased rate of amputation compared to those with normal values (adjusted HR: 3.71, 95% CI: 1.33–10.3).

## 4 DISCUSSION

The prognostic role of cardiac troponin, a well‐established biomarker in daily cardiology practice was not comprehensively studied in symptomatic PAD. According to our meta‐analysis that included more than five thousand PAD patients, elevated troponins on admission were associated with an increased risk of all‐cause mortality and major adverse cardiovascular events during a follow‐up period of more than 2 years. In addition, troponin positivity was more frequently detected in patients with CLI compared to those with intermittent claudication.

Due to the systemic nature of atherosclerosis, multisite artery disease is common in patients with PAD and is associated with worse clinical outcomes.^3^ However, screening for other sites of atherosclerosis (e.g., CAD) to improve outcomes in patients with lower extremity PAD is still a matter of debate.^2^ Therefore, a simple biomarker, as high sensitivity troponin, might identify individuals with silent myocardial injury, enable timely diagnosis and treatment and consequently improve the prognosis of vascular patients.

In the Atherosclerosis Risk in Communities study during a follow‐up period of over 22 years, high‐sensitivity cardiac troponin was independently associated with future risk of PAD, and particularly with its severe form CLI, independently of other atherosclerotic risk factors.^18^ The current work extends this knowledge, showing that cardiac troponins besides being useful for the diagnosis of incident PAD provide independent prognostic information.

CLI, which is the most severe manifestation of PAD, carries extensively high risk for unfavorable outcomes reflecting the systemic atherosclerotic burden associated with the disease.^2^ Hikita et al.^19^ performed coronary angiography on 242 symptomatic PAD patients and found greater CAD severity and higher high sensitivity troponin values in the CLI group, comparing to patients with intermittent claudication. In our meta‐analysis more than half of patients with CLI had elevated troponin levels, suggesting an extremely vulnerable subgroup of individuals with PAD. Moreover, Szczeklik et al.,^10^ in their population of CLI patients undergoing endovascular revascularization, showed that 85% of patients with positive troponin values did not have any ischemic clinical symptoms or electrocardiogram (ECG) changes at rest. In a study of patients with Fontaine stage II PAD undergoing treadmill testing, elevated baseline high sensitivity troponin levels were significantly associated with the risk of developing an exercise‐induced myocardial ischemia, that is, significant ST segment depression (≥0.2 mV).^20^ Moreover, elevated postoperative troponins were strongly associated with worse survival and greater likelihood of MACE after vascular surgery, regardless of whether symptoms of myocardial ischemia were present.^21^

Although a significant proportion of PAD patients have increased baseline cardiac troponin values, ischemic chest pain symptoms or baseline ECG changes are frequently lacking. This may be due to claudication or chronic leg pain at rest with limited ability to walk, and frequently present diabetes with subsequent neuropathy and silent coronary ischemia.^22^ Nowadays, high sensitivity cardiac troponins enable detection of subtle myocardial injury thus helping in diagnosing of underlying cardiac conditions, and not only related to CAD.^4^ Besides myocardial ischemia, an increased wall tension and ventricular strain due to long‐standing hypertension, heart valve disease and heart failure or kidney dysfunction and impaired renal clearance may contribute to increase troponin levels and poor prognosis.

Based on our findings, this may gain added value in risk stratification of this vulnerable population at extremely high cardiovascular risk. Moreover, patients with the most severe manifestation of PAD, that is CLI, may benefit the most.

In a recent meta‐analysis of plasma biomarkers and cardiovascular outcomes in PAD patients, among others, the role of cardiac troponins on all‐cause mortality was analyzed.^23^ It comprised four studies and unadjusted risk ratios were included in the analyses. Our meta‐analysis included eight high‐quality observational studies with adjusted HRs and reported pooled estimates of both all‐cause mortality and MACE. In addition, meta‐analyses of patients with CLI were performed.

Among patients with PAD in our meta‐analysis the overall prevalence of CAD was 57%, what is in accordance with the previous epidemiological data.^2^ In the vast majority of studies (seven out of eight) the effect estimates of Cox regression analyses were adjusted for CAD as a confounding variable. Also, in five out of eight studies recent acute coronary syndrome before enrollment or other acute cardiac diseases that may cause an increase of cardiac troponins were clearly indicated as exclusion criteria.^11^, ^13^, ^14^, ^16^, ^17^ In addition, risk of bias was evaluated for each study according to ROBINS‐I tool that included bias due to confounding. Two studies included in our systematic review reported receiver operating characteristic analysis for optimal cardiac troponin cutoff values in predicting adverse outcomes. In a study of patients with CLI Cimaglia et al.^17^ reported cardiac troponin cutoff of >40 ng/L for the prediction of MACE and >42 ng/L for all‐cause mortality. Clemens et al.^15^ in a population of claudicants identified the optimal troponin cutoff value of 9 ng/L for the prediction of mortality. In the West of Scotland Coronary Prevention Study with a follow‐up of 15 years, high concentrations of cardiac troponins (≥5.2 ng/L) were associated with an increased risk of coronary heart disease and cardiovascular death in middle‐aged hypercholesterolemic men without a history of myocardial infarction.^24^ Of note, cardiac troponin concentrations were reduced by statin treatment and those reductions were associated with better outcomes independent of LDL cholesterol lowering. In our meta‐analysis, overall 80% of PAD patients were on statin therapy. The oldest study that was included in our meta‐analysis^11^ reported the lowest use of statins (26%). However, Spark et al.^11^ in their study adjusted for statin treatment (among other confounders) in the multivariate analysis of factors predicting adverse outcomes. Unfortunately, patients with PAD still receive suboptimal medical therapy (including statins) when compared to patients with CAD. This may be due to the clinical inertia, atypical symptoms of PAD, or a focus on limb‐related rather than cardiovascular outcomes. The recent guidelines recommend statin therapy for all patients with PAD on the basis of reduced cardiovascular morbidity and mortality (Class 1 recommendation).^2^ Moreover, a recent meta‐analysis with 138 060 PAD patients (35% were treated with statins) showed that statin treatment reduced major adverse limb events by 30% and amputations by 35%.^25^

## 5 CONCLUSION

Troponin positivity is common in symptomatic PAD patients, especially in those with CLI. Elevated cardiac troponins on admission are independently associated with an increased risk of MACEs and all‐cause mortality in symptomatic peripheral vascular disease during a long‐term follow‐up.

## CONFLICT OF INTERESTS

The authors declare that there are no conflict of interests.

## Supporting information

Supporting information.

## Data Availability

The data that support the findings of this study are openly available in (MEDLINE/PubMed) at https://www.ncbi.nlm.nih.gov/pmc/, references.^10^, ^11^, ^12^, ^13^, ^14^, ^15^, ^16^, ^17^
