# Supplementary material for: Cardiac troponins predict mortality and cardiovascular outcomes in patients with peripheral artery disease: A systematic review and meta‐analysis of adjusted observational studies
Source: Clin Cardiol. 2022 Feb 7;45(2):198–204. doi: 10.1002/clc.23776 (PMC8860477; doi:10.1002/clc.23776)
Supplement: Supplementary file 1 — Supporting information. [file CLC-45-198-s001.docx]

**Supplementary TABLE** ROBINS-I tool for assessing risk of bias in included studies

| **Study** | **Bias due to confounding** | **Bias in selection of participants into the study** | **Bias in classification of interventions** | **Bias due to deviations from intended interventions** | **Bias due to missing data** | **Bias in measurement of outcomes** | **Bias in selection of the reported result** | **Overall Bias** |
| --- | --- | --- | --- | --- | --- | --- | --- | --- |
| **Spark, 2010** | **Low** | **Low** | **Low** | **Low** | **Low** | **Low** | **Low** | **Low** |
| **Linnemann, 2014** | **Moderate** | **Low** | **Low** | **Low** | **Low** | **Low** | **Low** | **Moderate** |
| **Pohlhammer, 2014** | **Moderate** | **Low** | **Low** | **Low** | **Low** | **Low** | **Moderate** | **Moderate** |
| **Otaki, 2015** | **Low** | **Low** | **Low** | **Low** | **Low** | **Low** | **Low** | **Low** |
| **Eisen, 2017** | **Low** | **Low** | **Low** | **Low** | **Low** | **Low** | **Low** | **Low** |
| **Szczeklik, 2018** | **Moderate** | **Low** | **Low** | **Low** | **Low** | **Low** | **Moderate** | **Moderate** |
| **Clemens, 2019** | **Moderate** | **Low** | **Low** | **Low** | **Low** | **Low** | **Moderate** | **Moderate** |
| **Cimaglia, 2021** | **Low** | **Low** | **Low** | **Low** | **Low** | **Low** | **Low** | **Low** |
